# Editorial: Natural products and nanoparticles in cancer treatment: is there a light at the end of the tunnel?

**DOI:** 10.3389/fphar.2024.1484339

**Published:** 2024-09-03

**Authors:** Milica Pešić, Amir Modarresi Chahardehi, Javier Echeverria, Wamidh H. Talib

**Affiliations:** ^1^ Institute for Biological Research “Siniša Stanković” – National Institute of the Republic of Serbia, University of Belgrade, Belgrade, Serbia; ^2^ Kimia Andisheh Teb Medical and Molecular Laboratory Research Co., Tehran, Iran; ^3^ Departamento de Ciencias del Ambiente, Facultad de Química y Biología, Universidad de Santiago de Chile, Santiago, Chile; ^4^ Faculty of Allied Medical Sciences, Applied Science Private University, Amman, Jordan

**Keywords:** natural products, nanoparticles, anticancer drugs, cancer treatment, cancer drug resistance

Cancer is a highly complex disease affecting a large global population, prompting ongoing exploration for safe and effective treatments. Natural products have been historically utilized for medicinal purposes, showing potential in cancer therapy. However, their clinical application is often hindered by challenges related to poor solubility, bioavailability, and stability. Nanoparticles are being studied for their unique properties that allow precise targeting of tumors and reduced impact on healthy tissues. Additionally, nanoparticles can be modified to target specific cancer cell receptors, improving targeting specificity. This dual targeting capability enhances the effectiveness of anticancer treatments and allows for combination therapies, where nanoparticles can deliver multiple therapeutic agents for synergistic effects. Incorporating natural product-derived compounds into nanoparticles can enhance therapeutic efficacy by improving drug delivery and stability and reducing toxicity.

Three articles within our Research Topic thoroughly explore the application of nanoparticles for targeted therapy across different types of cancer. The articles delve into the potential of nanoparticles to induce ferroptosis in cancer cells (Adzavon et al.), treat multiple myeloma (Smith et al.), and modulate macrophage phenotype to improve treatment outcomes in melanoma (Dossou et al.) One article is dedicated to natural products targeting angiogenesis in gynecological cancers (Jia et al.).

The emerging field of ferroptosis, an iron-dependent form of regulated cell death, holds great promise for overcoming therapy resistance in cancer treatment. A review paper by Adzavon et al. discusses ferroptosis resistance in cancer cells and explores the potential use of nanoparticles for combination therapy. It provides detailed insights into ferroptosis mechanisms in cancer cells, including iron metabolism, accumulation and regulation, reactive oxygen species generation, lipid peroxidation, glutamate synthesis, and the role of GPX4. The paper also discusses various ferroptosis resistance mechanisms, such as SLC7A11/GPX4, lipid peroxidation, FSP1/CoQ10 axis, the mevalonate pathway, DHOHD/CoQ2 axis, GCH1/BH4 axis, cholesterol metabolism, sex hormones, Wnt/ß-catenin pathway, and the tricarboxylic acid cycle. Furthermore, it explores the use of nanoparticles for inducing ferroptosis in cancer therapy, covering different types of nanoparticles including metal and metal-free nanoparticles. The paper emphasizes the development of nanoparticles that can promote cell death and stimulate an immune response. The review also highlights the importance of addressing challenges associated with ferroptosis resistance in cancer cells and calls for further research into resistance mechanisms and the identification of predictive biomarkers.

Smith et al. have been investigating a novel approach for addressing drug resistance in multiple myeloma (MM) patients. They have explored the use of caffeic acid phenethyl ester (CAPE) encapsulated in anti-biofouling iron oxide nanoparticles (IONP) as a potential alternative treatment. The encapsulation of CAPE in IONP has demonstrated stability in physiological conditions and controlled release in acidic environments, indicating its potential for targeted drug delivery to tumor sites. Additionally, the development of IONP with MM-cell-targeted ligand (RGD) for delivering CAPE has shown promise for image-guided drug delivery using T2-weighted MRI. Furthermore, the RGD-IONP/CAPE nano-formulation has exhibited enhanced inhibitory and apoptotic effects on MM cells *in vitro*, suggesting its potential as an effective and targeted therapeutic approach against MM with minimal impact on normal human cells. However, the interaction between bone marrow stromal cells and MM cells in co-culture has been observed to confer protection against the cytotoxic effects of CAPE, IONP/CAPE, and RGD-IONP/CAPE, indicating a potential limitation in the tumor microenvironment. Future research aims to explore strategies for overcoming this protective effect to enhance the effectiveness of RGD-IONP/CAPE and similar treatments.

The effectiveness of immunotherapies for advanced melanoma is limited by the highly immunosuppressive tumor microenvironment (TME), which obstructs immune cell function. Tumor-associated macrophages (TAMs) play a significant role in this immunosuppression. Dossou et al. investigated mannose-functionalized reconstituted high-density lipoprotein (rHDL) nanoparticles as a delivery system to reprogram TAMs. The results showed that the rHDL nanoparticles, loaded with the stimulator of interferon genes agonist DMXAA, decreased the levels of M2 markers. Through the mannose component, the rHDL nanoparticles enhanced the production of interferon β and CXCL10 compared to free DMXAA in the B16-F10 CM-educated RAW 264.7 macrophages. The mannose-functionalized rHDL nanoparticles delivered their payload (DMXAA) more effectively to the B16-F10 CM-educated RAW 264.7 macrophages than their non-mannosylated counterparts. The study findings demonstrate that incorporating the mannose component into the rHDL nanoparticles enhances payload delivery to the B16-F10 CM-educated macrophages via SR-B1 and CD206. The mannose-functionalized rHDL nanoparticles can reprogram macrophages to an M1 phenotype and increase sensitivity to PTX in B16-F10 melanoma cells. These findings indicate that the mannose-functionalized rHDL nanoparticles could improve TAM targeting and treatment outcomes when combined with immunotherapy or PTX.

In a comprehensive review by Jia et al., 63 natural products were identified as inhibiting angiogenesis in gynecological cancers. The authors categorized the mechanisms of natural products targeting angiogenesis in gynecological cancers into those involving signaling pathways of VEGF, PI3K/AKT, HIF-1α, or ERK through comparative analysis. The primary target for impeding tumor neovascularization was the VEGF pathway. While most of the research data are derived from *in vitro* and *in vivo* experiments, there is limited data from clinical trials. Despite the significant potential of natural products in treating gynecologic cancers, concerns remain regarding specific side effects. Increasing dosing frequency to maintain optimal efficacy may lead to reduced patient adherence and cumulative drug toxicity. Furthermore, some natural products have limited oral bioavailability, particularly oily components derived from plants. However, there have been developments in drug delivery systems, such as nanoemulsions, to enhance their biocompatibility and accessibility.

In summary, the current Research Topic has generated a high level of interest, but there are still important aspects that need to be addressed. These include examining the impact of nanoparticles and natural products on passage through physiological membranes, delivery to protected organs such as the brain, and their anticancer effects at tumor sites. We anticipate new studies to explore these areas and demonstrate the potential of nanoparticles and natural products in treating different types of tumors. Using nanoparticles and natural products to minimize the toxicity of cancer chemotherapy could lead to better tolerated and more effective treatments.

